# Cardiac morbidity in HIV infection is associated with checkpoint inhibitor LAG-3 on CD4 T cells

**DOI:** 10.1371/journal.pone.0206256

**Published:** 2018-10-31

**Authors:** Suresh Pallikkuth, Rajendra Pahwa, Bagavathi Kausalya, Shanmugam Saravanan, Li Pan, R. Vignesh, Syed Iqbal, Sunil S. Solomon, Kailapuri G. Murugavel, Selvamuthu Poongulali, Nagalingeswaran Kumarasamy, Savita Pahwa

**Affiliations:** 1 University of Miami Miller School of Medicine, Miami, Florida, United States of America; 2 YRG Centre for AIDS Research and Education (YRG CARE), Chennai, India; 3 Johns Hopkins University School of Medicine, Baltimore, MD, United States of America; Uniformed Services University, UNITED STATES

## Abstract

Recent findings point to a role of Checkpoint Inhibitor (CPI) receptors at the tissue level in immune homeostasis. Here we investigated the role of CPI molecules on immune cells in relation to cardiac function. Participants recruited in Chennai, India consisted of HIV+ ART naive viremic (Gp1 n = 102), HIV+ on ART, virologically suppressed (Gp2, n = 172) and HIV negative healthy controls (Gp3, n = 64). A cross-sectional analysis of cardiac function, arterial resistance and immunologic assessment of CPI expressing T cells was performed. Data indicate that ART naive exhibited cardiac function impairment and greater arterial stiffness than the other groups. Frequencies of CD4+ T cells expressing LAG-3 and PD1 were higher in ART naïve while TIGIT and TIM3 were similar among the patient groups. LAG-3+, PD1+ and dual LAG-3+PD1+ CD4 T cells were inversely correlated with cardiac function and arterial elasticity and directly with arterial stiffness in ART naïve participants and with arterial elasticity in virally suppressed group on ART. We conclude that HIV induced upregulation of LAG-3 singly or in combination with PD1 in immune cells may regulate cardiac health and warrant mechanistic investigations. The implications of these findings have bearing for the potential utility of anti-LAG-3 immunotherapy for cardiac dysfunction in chronic HIV infection.

## Introduction

Cardiovascular disease (CVD) is a major contributor to mortality and morbidity in HIV infection, and is largely attributed to underlying inflammation and immune activation (IA) which is known to persist, albeit at a lower level following antiretroviral therapy (ART) [1, 2]. Early in the era of ART, the drugs themselves were found to be cardiotoxic, but this issue is now considered of less relevance with newer drugs that have minimal or no cardiac toxicity [3]. Persistent T cell activation in chronic HIV infection leads to a chronic inflammatory environment that has multiple deleterious effects at the tissue level, directly or indirectly inflicting damage to different organ systems, the mechanisms of which are not well understood. Immune activation at the cellular level, that involves CD4 and CD8 T cells results in T cell proliferation and dysfunction [4, 5]. Intrinsic mechanisms that maintain T cell numbers at a constant level do so by balancing immune activation and homeostatic proliferation. These mechanisms include regulation of cell death molecules such as Fas/FasL [6, 7] and immune checkpoint inhibitor (CPI) molecules such as Programmed cell death protein 1 (PD1), Lymphocyte-activation gene 3 (LAG-3), T cell immunoglobulin and mucin domain 3 (TIM3), T cell immunoreceptor with Ig and ITIM domains (TIGIT) and cytotoxic T-lymphocyte-associated protein4 (CTLA-4) [8–10]. In lymphocytes, the CPI have critical roles in the maintenance of immune homeostasis by ensuring contraction of effector T cell responses [11, 12] and protects the host from exuberant anti-microbial responses. The expression of LAG-3, TIGIT and CTLA-4 on T regulatory cells (Tregs) enable the Tregs to suppress effector T cell function [13–17]. In acute HIV infection as well, CPI may serve to protect the host from end organ damage, and may be cardio-protective. In contrast to acute infection, in chronic untreated HIV infection and malignant states however, chronic antigen stimulation can lead to sustained immune activation and inflammation resulting in elevated expression of CPI molecules on effector T cells with dampened immunity manifesting as functional unresponsiveness of the immune system [18, 19] and reduced effector function of CD4 and CD8 T cells [8, 20, 21]. Together these effects may lead to end organ damage that potentially could be rescued by effective ART as shown in the present study.

While all CPI are considered in general terms as having an immunoregulatory role, they may have unique properties and distinct mechanisms of action [8, 22–24]. For example co-inhibitory receptors CTLA-4 and PD1 are primarily responsible for maintaining self tolerance by restricting T cell clonal proliferation in lymphoid organs while LAG-3, TIM3, and TIGIT have been assigned specific roles related to regulation of tissue inflammation [8, 16, 25–31]. Specificity for the regulatory activity of the CPI resides at the tissue level, based on the ligands expressed on tissues that maintain tissue tolerance and inhibit immunopathology. In fact, there is evidence for the expression of the MHC class II, the receptor for LAG-3 in cardiac endothelial cells and endocardial cells in certain disease states in humans and rodents [32–35] as well as in cultured human fetal cardiac myocytes in experimental systems [36].

The present study investigated the expression of CPI molecules on CD4 T cells in HIV+ ART naïve viremic and in virologically suppressed group on ART for their relationship to measures of cardiac function and arterial stiffness. Additionally, healthy individuals were investigated and served as controls.

## Materials and methods

### Study setting and subjects

This study was conducted at YRG CARE, a tertiary care center located at Chennai in South India, and enrolled 274 male and female subjects with chronic HIV infection (HIV+) and 63 HIV-uninfected healthy controls (HC) in the age range >18 yr-50 yr (**Table 1)**. Among the HIV+ participants, 102 were ART naïve (group 1) and 172 were on ART for >12 months (group 2) with viral suppression as determined by two consecutive plasma viral load values of <40 copies/mL. The 63 HC were categorized as group 3. Patients with pre-exposure or post-exposure ART prophylaxis and pregnant women were excluded. Distribution of participants was matched for age, race, gender, basal metabolic rate, smoking, and diet history. At enrollment, among ART naïve and ART treated virally suppressed HIV+ patients respectively 8% (8/101) and 13% (22/172) were current smokers, 67.5% (68/101) and 57% (98/172) had never smoked; 24.6% (25/101) and 30% (52/172) had smoked in the past. Only 2 in each group were HBV and HCV+. The study was approved by both YRG CARE and University of Miami institutional review boards. Informed consent was obtained from all enrolled participants. A detailed interview was conducted at the time of enrolment to collect demographic information. All participants had a one time blood draw for collection of plasma and peripheral blood mononuclear cells (PBMC). Blood samples were processed within an hour of collection according to guidelines of the AIDS Clinical Trials Group. Plasma was stored in aliquots at -80°C and PBMC were cryopreserved in liquid nitrogen in aliquots of 5 million cells/mL.

**Table 1:** Characteristics of the study cohort.

	Gp 1, ART Naive	Gp 2, Virologically Suppressed on ART	Gp 3 Healthy Controls	*P-* value
Number	102	172	63	-	
Gender, M/F	47/55	116/56	22/41		
Age (in years)	36.5 ±5.8	38.6 ±5.9	36.8 ±7.09		
Median duration of infection; (in mo.) (range)	15 (2–42)	72 (39.5–92)	NA	<0.001* (Gp1 vs Gp 2)	
Mean Nadir CD4+ T-cell count, cells/ μL; (range)	NA	312.3 **±** 199 (5–1425)	NA	__	
Mean CD4+ T-cell count at study entry; cells/ μL) ± SD (range)	421.4 **±** 321.6 (21–1897)	743.4 **±** 321.6 (154–1863)	NA	<0.001*	
Median Duration of ART in months; (range)	__	43 (22–72)	NA	__	
Body Mass Index (Kg/m^2^); Median (range)	22.7 (20.1–26.3)	22 (19–25.8)	24.6 (22.5–27.1)	0.013* (Gp1vs3) 0.006*(Gp 2vs3)	

All descriptive variables are provided as median and interquartile ranges except for age, Nadir CD4 and CD4 counts at entry which are provided as mean ± SD. T-test was used to calculate *p*-value between the groups for ‘age’ which was normally distributed, while Mann-Whitney U-test was used for other non-normally distributed variables.

* indicates statistical significance.

### Measures of cardiac function and arterial stiffness

Pulse rate, stroke volume, stroke volume index, cardiac output, cardiac index and cardiac ejection time were determined to ascertain cardiac functioning. Arterial stiffness was estimated by pulse-wave velocity (PWV) using the HDI/PulseWave CR-2000 (Hypertension Diagnostics, Inc., Eagan, MN), a diagnostic tool that was previously applied in the INSIGHT Strategic Timing of Anti Retroviral Treatment arterial stiffness sub-study [37]. Along with Large Artery Elasticity index (LAE) and Small Artery Elasticity index (SAE) measures, systemic vascular resistance (SVR) and total vascular impedance (TVI) were measured as arterial stiffness parameters.

### Flow cytometry for analysis of checkpoint inhibitor molecules and immune activation

Thawed PBMC were rested overnight and 1x10^6^ cells were stained with Live/Dead Aqua followed by staining for surface markers CD3, CD4, CD8, checkpoint inhibitors PD1, TIGIT, TIM3, and LAG-3 and immune activation markers HLA-DR and CD38. Cells were then fixed, and acquired on a Flow cytometer (BD LSRFortessa, San Jose, CA) and analyzed by FlowJo V10 (Treestar, Ashland, OR). Frequencies of CPI molecules either alone or in combinations were analyzed on live (Aqua-) CD3+CD4+ and CD3+CD8+ T cells. Immune activation was measured based on the dual expression of HLA-DR and CD38 on CD4 and CD8 T cells.

### Soluble LAG-3 in plasma by ELISA

Plasma levels of soluble LAG-3 (sLAG-3) were measured by ELISA (Abcam, Cambridge, MA) as per the manufacturer’s recommendations using a sample dilution of 1:5. Data are expressed as pg/ml.

### Statistical analysis

Descriptive statistics such as percentages, means and standard deviation, and median were used to describe the demographic characteristics of the study population. For unpaired data, Levene’s test was used firstly to check variance heterogeneity followed by Wilcoxon rank-sum test (also called ‘Mann-Whitney’ U test) was performed using R stats package. For Correlation analyses, Shapiro test was used to check if data are normally distributed using R stats package. If Shapiro test showed p>0.05, Pearson correlation coefficient was performed using R stats package; in other instances Spearman's rank correlation coefficient was performed using R stats package. A *p* value of <0.05 was considered as significant. The data are presented as scatter plots with regression lines and correlation coefficients with *P* values.

## Results

### Demographic characteristics of study population

Demographic characteristics of the study population are shown in **Table 1**. The mean ages were similar among the study groups. Median duration of infection was lower in the ART naïve compared to ART treated virologically suppressed group. Of the patients on ART, 140 patients were on first-line reverse transcriptase inhibitors (RTI; AZT/D4T/TDF+3TC/FTC+EFV/NVP) while 32 were on Protease Inhibitors (RTV-boosted LPV) based second-line therapy at the time of study enrolment. Absolute CD4 counts were significantly lower in the ART naive compared to virologically suppressed while BMI was significantly lower in both HIV+ groups compared to healthy controls.

### Checkpoint inhibitor molecule expressing T cells are increased in HIV+ individuals

**Fig 1:**
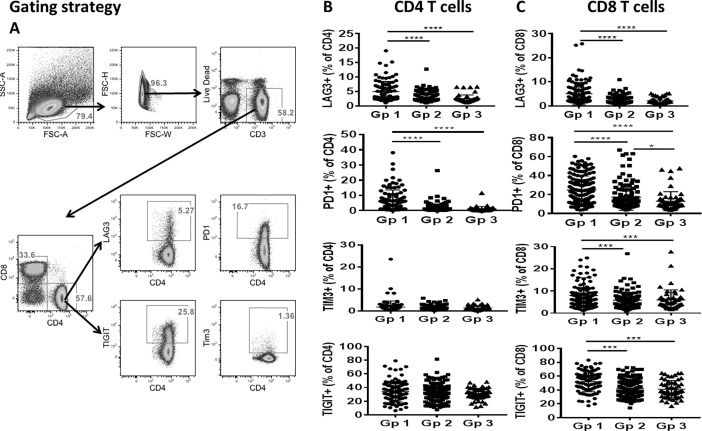
Higher frequencies of LAG-3, PD1, TIGIT and TIM3 on CD4 and CD8 T cells in ART naïve group. Checkpoint inhibitor (CPI) molecules and T cell immune activation markers were analyzed on CD4 and CD8 T cells by flow cytometry in ART naïve (Gp1) and virologically suppressed (Gp2) patients and healthy controls (Gp3). A), Representative flow cytometry dot plots showing gating strategy for the analysis of CPI on CD4 T cells. Frequencies of LAG-3+, PD1+, TIGIT+, and TIM3+ cells are shown in B), for CD4 T cells and C), for CD8 T cells. Line and error bars within the plot indicate the Mean ± SD. Data compared between groups using Wilcoxon rank-sum test. A p value <0.05 was considered significant. *p<0.05; **p<0.01; ***p<0.001; ****p<0.0001.

We analyzed the expression of CPI molecules LAG-3, PD1, TIGIT and TIM3 on CD4 and CD8 T cells in the study groups. Flow cytometry gating strategy for analysis of the CPI on CD4 T cells is depicted in **Fig 1A**. Among CPI, as shown in **Fig 1B**, frequencies of LAG-3 and PD-1 on CD4 T cells and MFI (not shown) were significantly higher in ART naive compared to virologically suppressed on ART and healthy controls, but the frequencies and MFI (not shown) of TIGIT and TIM3 were not different between the study groups.

In the CD8 T cell compartment, both frequencies and MFI (not shown) of LAG-3, PD1, TIM3 and TIGIT were significantly higher in the ART naive compared to virologically suppressed group and healthy control group **(Fig 1C)**. Moreover, ART treated virologically suppressed group also showed higher frequencies of PD1+CD8 T cells compared to healthy control group **(Fig 1C)**.

**Fig 2:**
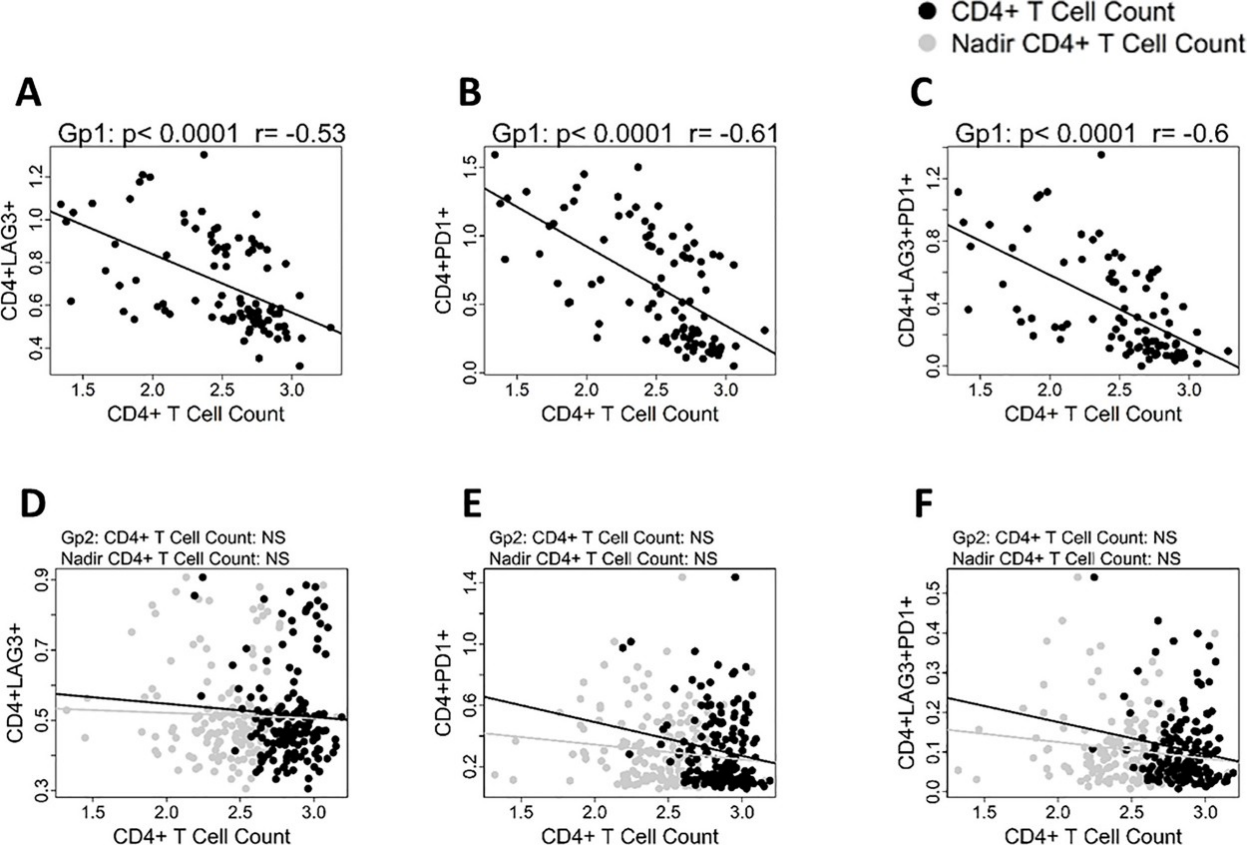
Absolute CD4 numbers correlate with CPI molecule expression on CD4 T cells in ART naïve individuals. A-C: ART naïve Gp1 patients. Linear regression analysis shows correlation between CD4 T cell counts at study entry with A), LAG-3+CD4; B), PD1+CD4 and C), LAG-3+PD1+ CD4 T cells. D-F: Virologically suppressed Gp2 patients. Linear regression analysis shows correlation between CD4 T cells count (black circles) and nadir CD4 T cells (grey circles) with D), LAG-3+CD4; E), PD1+CD4 and F), LAG-3+ PD1+ CD4 T cells. Pearson correlation was performed based on data distribution; a *p* value of <0.05 was considered as significant.

Absolute numbers of CD4 T cells at study entry inversely correlated with LAG-3, PD1 and with dual LAG-3+PD1+ CD4 T cells in ART naïve group (**Fig 2A and 2B and C, respectively**). In the virologically suppressed group, no correlation was observed of absolute CD4 T cell counts or of nadir CD4 T cell counts with LAG-3 or PD1 or dual LAG-3+PD1+ CD4 T cells **(Fig 2D, 2E and 2F, respectively).**

We also measured soluble form of LAG-3 in plasma from a subset of participants from each of the three groups, **(S1 Fig)**. Levels of sLAG-3 were significantly higher in the ART naïve group as compared to virologically suppressed- and healthy control groups, which were not different from each other.

### CD4 and CD8 T cell immune activation in ART naïve group correlates with LAG-3 and PD1 expression on CD4 and CD8 T cells

**Fig 3:**
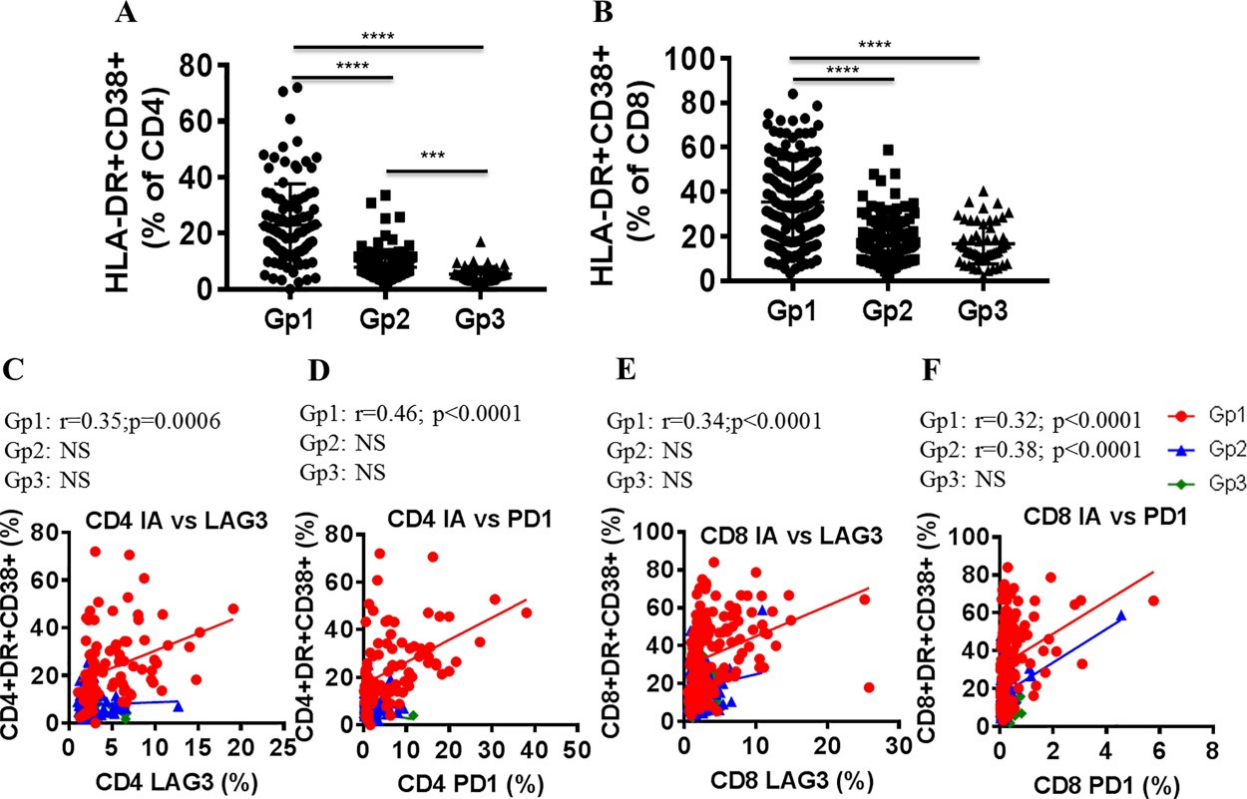
LAG-3 and PD1 expression on CD4 and CD8 T cells correlate with T cell immune activation in ART naïve groups. Immune activation on CD4 and CD8 T cells was measured based on the co-expression of HLA-DR and CD38 by flow cytometry. Frequencies of HLA-DR+CD38+ expressing A), CD4 and B), CD8 T cells are shown. Linear regression analysis shows correlation between CD4 T cell immune activation with C), LAG-3+ and D), PD1+ CD4 T cells and of CD8 T cell immune activation with E), LAG-3+ and F), PD1+ CD8 T cells in ART naïve (Gp1, red circles), virologically suppressed (Gp2, blue circles) and healthy control (Gp3, green circles) groups. Pearson correlation was performed based on data distribution; a *p* value of <0.05 was considered as significant.

Analysis of CD4 and CD8 T cell immune activation showed higher frequencies of HLA-DR+CD38+ CD4 and CD8 T cells in the ART naïve group compared to ART treated virologically suppressed and healthy control groups **(Fig 3A and 3B)**. In addition, the virologically suppressed group showed higher frequencies of HLA-DR+CD38+ CD4 T cells compared to the healthy control group **(Fig 3A)**. Both CD4 and CD8 T cell immune activation correlated with LAG-3 and PD1 expression on CD4 and CD8 T cells in ART naïve group **(Fig 3C–3F)**. CD8 T cell immune activation in virologically suppressed group on ART also showed direct correlation with PD1+CD8 T cells **(Fig 3F)**.

### Cardiac function and arterial stiffness are impaired in ART naïve group

**Fig 4:**
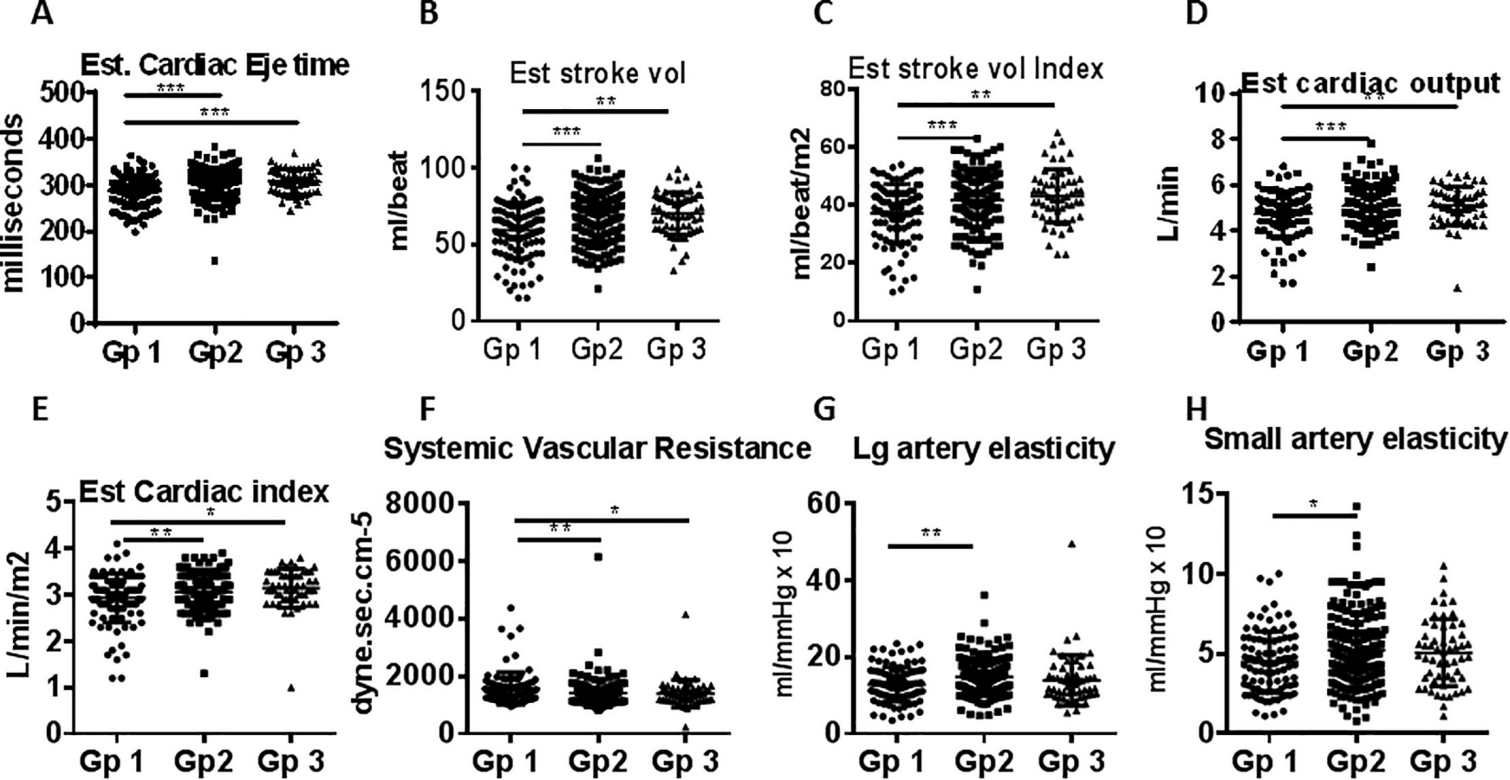
Cardiac functions are impaired in HIV+ ART naive group. Cardiac function and arterial stiffness related measures were compared between ART naïve (Gp1) and virologically suppressed on-ART (Gp2) patients and healthy controls (Gp3). Measures of cardiac functions include A), estimated cardiac ejection time; B), estimated stroke volume; C), estimated stroke volume index; D), estimated cardiac output and E), estimated cardiac index. F-H: Measures of arterial stiffness include F), systemic vascular resistance, G), large artery elasticity index and H), small artery elasticity index. Data between groups are compared using Wilcoxon rank-sum test; a p value of <0.05 was considered significant. *p<0.05; **p<0.01; ***p<0.001; ****p<0.0001.

Measures of cardiac function and arterial stiffness in the study groups are shown in Fig 4. The ART naïve had lower cardiac ejection time **(Fig 4A)**, lower stroke volume **(Fig 4B),** lower stroke volume index **(Fig 4C),** lower cardiac output (**Fig 4D),** and lower cardiac index **(Fig 4E)** compared to virologically suppressed on ART and healthy controls. Measures of arterial stiffness showed higher systemic vascular resistance **(Fig 4F)** with lower large and small artery elasticity **(Fig 4G and 4H respectively)** in ART naive compared to virologically suppressed group. Cardiac function and arterial stiffness parameters were not different between virologically suppressed group and healthy controls. These data support an HIV induced effect on multiple cardiac functions and on arterial stiffness in ART naïve HIV+ group. In the virologically suppressed group, these parameters did not differ significantly from healthy controls implying ART- mediated improvement.

### Correlation of cardiac function and arterial stiffness with LAG-3+ CD4 T cells

**Fig 5:**
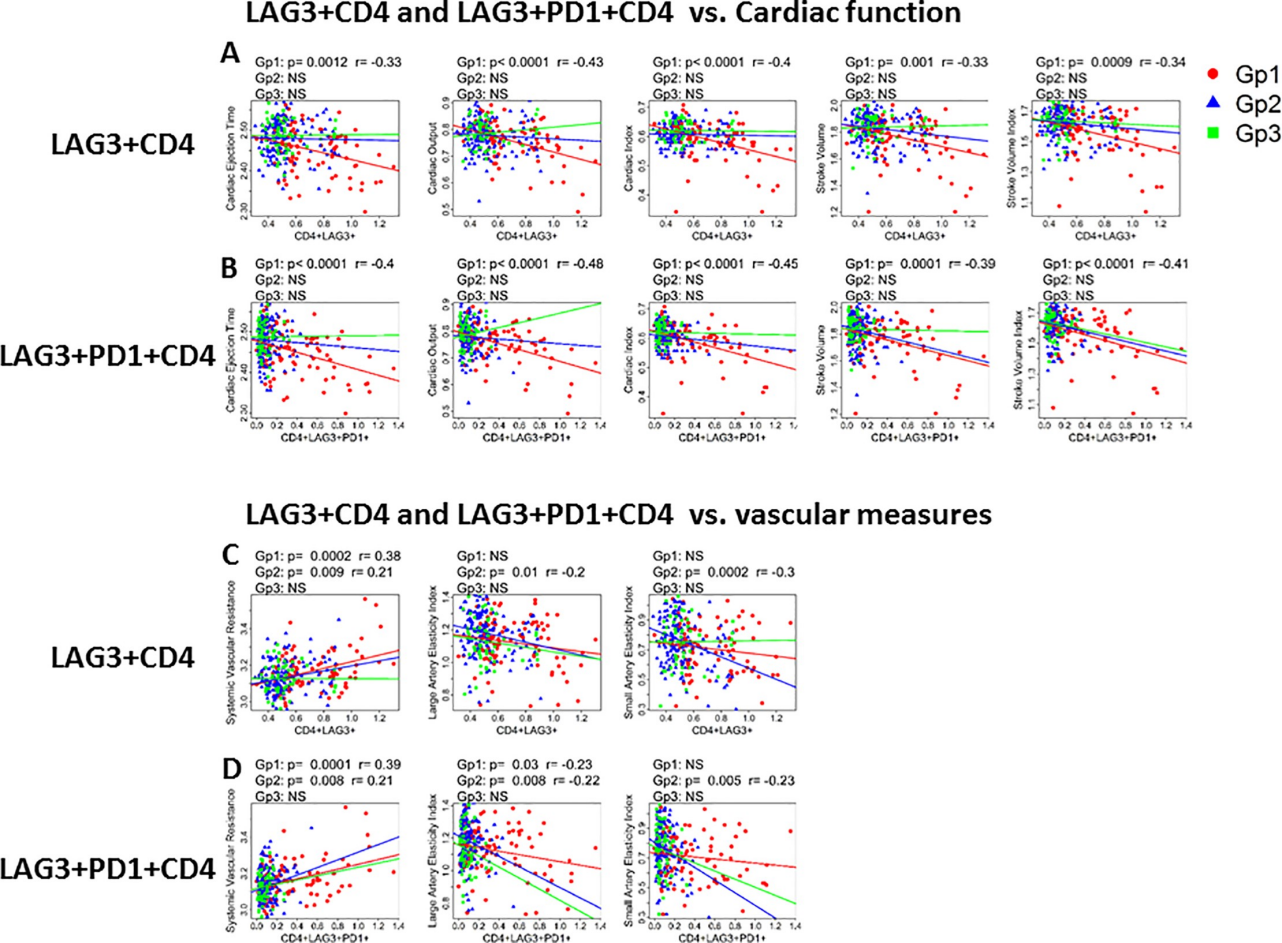
Markers of cardiac function and arterial stiffness correlate with CPI expression on CD4 T cells. Linear regression analysis showing correlation of cardiac function with A), LAG-3+ CD4 T cells; B), LAG-3+PD1+ CD4 T cells. Correlation of arterial stiffness with C), LAG-3+CD4 and D), LAG-3+PD1+ CD4 T cells shown for ART naïve (Gp1, red), Virologically suppressed (Gp2, blue) and healthy controls, (Gp3, green). Pearson correlation was performed based on data distribution; a *p* value of <0.05 was considered as significant.

We investigated the relationship of CPI molecules on measures of cardiac function. Linear regression plots for association of cardiac function measures (**Fig 5A and 5B)** showed that in ART naïve group, single LAG-3 or LAG-3 plus PD1 expressing CD4 T cell subsets respectively were inversely correlated with measures of cardiac ejection time, cardiac output, cardiac index, stroke volume, and stroke volume index. In the virologically suppressed and healthy control groups, no association of LAG-3 or LAG-3 plus PD1 expressing CD4 T cells with measures of cardiac function was evident **(Table 2).** In addition to CD4 T cells, LAG-3, PD1, and LAG-3 plus PD1 expressing CD8 T cells, also showed a weak inverse correlation with markers of cardiac function in ART naïve group **(Table 3).**

**Table 2:** Correlations between LAG-3, PD1 and LAG-3+PD1+ expressing CD4 T cells and cardiac function.

Cardiac function	CD4+LAG 3+ (%)		CD4+PD1+ (%)		CD4+LAG 3+ PD1+ (%)	
Gp1 (ART Naïve)	p value	r value	p value	r value	p value	r value
Cardiac ejection time	0.001	-0.33	0.001	-0.33	<0.0001	-0.4
Cardiac output	<0.0001	-0.38	0.001	-0.33	<0.0001	-0.42
Cardiac index	<0.0001	-0.4	<0.0001	-0.39	<0.0001	-0.45
Stroke volume	0.001	-0.33	0.005	-0.29	<0.0001	-0.39
Stroke volume index	<0.0001	-0.34	0.001	-0.33	<0.0001	-0.41
Systemic vascular-resistance	0.0002	0.38	0.0005	0.36	0.0001	0.39
Large artery elasticity	NS	-	NS	-	0.03	-0.23
**Gp2 (Virologically suppressed)**						
systemic vascular-resistance	0.009	0.21	0.03	0.17	0.008	-0.21
Large artery elasticity	0.009	**-0.21**	0.01	-**0.21**	<0.0001	-0.19
Small artery elasticity	0.001	**-0.26**	NS	-	0.03	-0.17

Cardiac function and vascular measures were correlated with LA-G3+, PD1+ and LAG-3 plus PD1+ CD4 T cells using Pearson correlation. Data are shown for correlation coefficient (r) and significance (p). A p value of <0.05 was considered significant.

**Table 3:** Correlations between LAG-3, PD1 and LAG-3+PD1+ expressing CD8 T cells and cardiac function.

Cardiac function	CD8+LAG 3+ (%)		CD8+PD1+ (%)		CD8+LAG 3+PD1+ (%)	
Gp1 (ART Naïve)	p value	r value	p value	r value	p value	r value
Cardiac ejection time	0.023	-0.22	0.005	-0.28	0.0003	-0.36
Cardiac output	0.002	-0.30	0.017	-0.24	0.002	-0.31
Cardiac index	0.004	-0.28	0.013	-0.24	0.002	-0.31
Stroke volume	0.013	-0.24	0.007	-0.27	0.0006	-0.34
Stroke volume index	0.013	-0.28	0.004	-0.28	0.0005	-0.35
Systemic vascular-resistance	0.017	0.24	NS	-	NS	-
Large artery elasticity	NS	-	NS	-	0.004	-0.3
**Gp2 (Virologically suppressed)**						
systemic vascular-resistance	0.009	0.21	NS	-	NS	-
Large artery elasticity	0.021	**-0.17**	0.036	-0.16	NS	-
Small artery elasticity	0.001	**-0.25**	NS	-	NS	-

Cardiac function and vascular measures were correlated with LAG-3+, PD1+ and LAG-3+plus PD1+ CD8 T cells using Pearson correlation. Data are shown for correlation coefficient (r) and significance (p). A p value of <0.05 was considered significant.

Vascular measures of systemic vascular resistance directly correlated with LAG-3 **(Fig 5C)** or LAG-3+PD1+ CD4 T cells in ART naive and virologically suppressed groups **(Fig 5D)**. Large artery elasticity index inversely correlated with LAG-3+PD1+ CD4 T cells in Gp1 but with LAG-3 alone, or in combination with PD1 in Gp2. Inverse correlations of small artery elasticity index and single LAG-3 or LAG-3 co-expressed with PD1 on CD4 T cells were only found in virologically suppressed group. Single PD1 expressing CD4 T cells followed a similar pattern but were not consistent. A summary of correlations are depicted in a heatmap shown in **S2 Fig**. No associations were found between arterial stiffness measures and CPI in healthy controls **(Fig 5C and 5D).** Levels of sLAG-3 did not show a correlation with cardiac markers (data not shown).

## Discussion and conclusions

Cardiovascular disease is a major contributor to mortality and morbidity in HIV infection [3, 38–40]. This cross sectional study was aimed at defining relationship of checkpoint inhibitor molecules on T cells and CVD in chronic HIV infection. We conducted the study in ART naïve viremic patients as well as in patients on ART with viral suppression and healthy age-matched HIV uninfected controls. In the ART naïve group, cardiac function was decreased with evidence of increased vascular resistance. We observed that LAG-3, PD1 or LAG-3 plus PD1 expressing CD4 T cells were inversely correlated with cardiac function, while being directly correlated with vascular resistance. Virologically suppressed patients, despite having started ART at CD4 nadirs similar to ART naïve, had little evidence of impaired cardiac function but had residual increased vascular resistance involving large and small vessel elasticity, which was also inversely correlated with LAG-3 expressing CD4 T cells and maximally with cells co-expressing LAG-3 and PD1. These observations, together with the high LAG-3 expression in monocytes from healthy controls (unpublished observations), imply a dominant role of cells expressing LAG-3 alone or in combination with PD1, but without association of TIGIT or TIM3 in regulating cardiac health, particularly in viremic patients with chronic HIV infection who have not started ART. The benefits of ART on CVD and on expression of CPI on CD4 T cells were clearly manifest in this study based on comparisons of ART naïve- with ART treated and healthy control groups.

Recent evidence points to expression of receptors for CPI molecules/receptors at the tissue level for controlling organ immune homeostasis. The receptor for LAG-3 is the major histocompatibility complex Class II molecule. Caforio et at al investigated human cardiac tissue for expression of MHC Class II molecules [32]. These authors found that endothelial and endocardial cells expressed MHC Class II molecules in a majority of patients with dilated cardiomyopathy (DCM), and less frequently in other acquired cardiac diseases, congenital heart disease, or in normal hearts. The expression of MHC II molecule on cardiac endothelial and endocardial cells suggests a possible pathogenic role of these molecules in the initiation and/or perpetuation of DCM [32]. Activated endothelial cells may be involved in the homing and trafficking of lymphocytes [41, 42] in the affected tissue in the early stages of the disease. It is possible that a similar phenomenon occurs in HIV infection with MHC class II expression on cardiac endothelial and endocardial cells and trafficking of CD4+LAG3+ T cells to these sites. Only studies at the tissue level in ART naive and ART treated subjects can elucidate the pathology leading to cardiac dysfunction in HIV subjects.

LAG-3 is a 498-amino acid transmembrane protein identified on activated human NK and T cells [43]. The gene for LAG-3 in humans is located adjacent to CD4 on chromosome 12 and is structurally homologous to CD4 with four extracellular immunoglobulin superfamily like domains D1, D2, D3, and D4 [44–46]. In T cells, LAG-3 mRNA levels increase 10 fold upon cell activation [47] which is highly relevant as LAG-3 function is mediated by modulation of LAG-3 expression at the transcriptional level [46]. Intracellular storage in lysosomal compartments, may also serve to facilitate rapid LAG-3 cell surface expression following T cell activation [11, 12, 48, 49].

Upregulation of LAG-3 on T cells also defines a subpopulation with functional exhaustion that correlates with disease progression in HIV infected individuals (50). LAG-3 expression in T cells was significantly upregulated in HIV infected individuals and was correlated with disease progression. In 28 HIV infected individuals they analysed the relationship of LAG-3 to immune activation markers CD38 and HLA-DR and noted that LAG-3 was largely co-expressed with CD38 on 70% of CD4 T cells and 74% of CD8 T cells, while the percentage of cells co-expressing LAG-3 and HLA-DR was less. In that study, MFI of LAG-3 expression declined in both CD4 and CD8 T cells after more than one year of ART treatment [50]. LAG-3 is a natural high-affinity ligand for MHC class II molecules [8, 44, 45] and has an inhibitory role in regulating T cell immune responses [8, 46] and also acts synergistically with PD1 to regulate T cell function [46, 51]. Our findings are in agreement with these observations as we found that immune activation and LAG-3 and PD1 expressing CD4 T cells were greater in ART naïve viremic subjects than in those virally suppressed on ART where the lower LAG-3 expression is most likely a consequence of ART induced viral suppression and reduction in immune activation. Virologically suppressed participants were similar to healthy controls in both, the measures of CVD and frequency of CPI expressing CD4 T cells, supporting the beneficial effect of ART and virologic suppression on cardiac function. Although CPI molecules are associated with regulating immune activation, the direct association between T cell immune activation and CPI in the presence of viremia merits further investigation.

Risk factors for CVD are under intense investigation and involve both traditional and non-traditional factors. A study by Golden et al [52] found that the lipoprotein scavenger receptor class B type 1 (SCARBI) rs10846744 noncoding variant is significantly associated with atherosclerotic disease independently of traditional cardiovascular risk factors. They identified a connection between rs10846744 with sLAG-3 in plasma and concluded that plasma sLAG-3 is an independent predictor of HDL-cholesterol levels and CVD risk. We did not find a correlation of sLAG-3 with cardiac measures in our study participants, but cannot rule out the possibility that it is a player in mediating tissue damage to the heart. In murine studies, sLAG-3 has not been found to have any biologic function [47, 53]. Soluble LAG-3 is released by cell surface shedding and is cleaved by the metalloproteinases ADAM10 and ADAM17, which cleave a wide range of transmembrane proteins including CD62L, TIM3 and TNFα [54]. The action of ADAM17 becomes evident following cell activation [11, 12, 48, 49], and sLAG-3 has been reported to be elevated in humans in situations of T cell activation, as in infection or autoimmunity [55–57]. In our study sLAG-3 in plasma followed a similar pattern as surface LAG-3 and the levels of sLAG-3 were higher in ART naïve group compared to ART treated or healthy controls. The biologic relevance of sLAG-3 in the context of cardiac disease in HIV is unclear.

It is important to understand the physiological role of individual CPI molecules in terms of their independent effects and dependence upon each other. Although they belong to the same class of receptors, different CPI often act in a tier fashion, with CTLA-4 and PD1 in the first tier for maintaining self-tolerance and LAG-3, TIM3 and TIGIT in a second tier with distinct roles in regulating immune responses particularly at sites of tissue inflammation [8, 16, 25–31]. In a murine transplant tumor model, it was observed that PD1 and LAG-3 were coexpressed on tumor-infiltrating CD4 and CD8 T cells at the tissue level and also in cancer patients [49, 58–60]. In our study PD1 and LAG-3 coexpression on CD4 T cells was highly correlated with cardiac disease measures and significance was often greater than that of independent molecules, implicating their involvement in regulation of organ systems at tissue levels.

The inference about the potential involvement of PD1 on CD4 T cells in influencing cardiac function was based on its higher expression in ART naive patients compared to other groups and data of the correlation analyses with cardiac measures. PD1 is an inhibitory receptor expressed by activated T, B and myeloid cells that was discovered by the Nobel laureate Tasuku Honjo in 1992 [61], who later with Freeman et al discovered its ligand PD-L1 as a member of the B7 gene family [62]. Engagement of PD1 by PD-L1 leads to the inhibition of T cell receptor-mediated lymphocyte proliferation and cytokine secretion [62]. Nishimura et al demonstrated the occurrence of autoimmune dilated cardiomyopathy and cardiac dysfunction in PD1 receptor deficient mice with diffuse deposition of IgG on the surface of cardiomyocytes and high titer circulating Ig-G autoantibodies [63]. Cardiomyocytes were found to be a major source of inflammatory cytokine generation in experimental systems using isolated ischemic-reperfused hearts [64–66]. Importantly, PD1 and PD-L1 were found to be expressed on cardiomyocytes and on different populations of cardiac cells. Upregulation of both PD1 and PD-L1 has been demonstrated in an acute cardiac injury model of ischemic-reperfused and cryoinjured hearts [67]. In this study PD1/PD-L1 pathway has been postulated to play an important role in cardiac injury by increasing GADD153, a regulator of the inflammatory response, leading to pro-inflammatory changes in the ischemic refused hearts with a marked increase of IL-17+ cells and only a mild increase in IL-10+ cells. Furthermore, PD-L1 blocking antibody treatment reduced cardiac GADD153 expression with reduction in expression of inflammatory cytokines [67]. It is possible that LAG-3 and PD1 contribute towards immune dysfunction at the local tissue level within the heart muscle microenvironment as ligands for both can be expressed on cardiac myocardium endothelial cells and LAG-3 plus PD1 coexpressing immune cells may inflict maximal cardiac damage.

The impact of CPI on immune cells in tissues is most likely influenced by state of virological control and severity of HIV disease status. Our data indicate that ART induced virus suppression in chronic HIV infection may lead to better cardiac function and reduced arterial stiffness together with decrease in immune activation as demonstrated by fewer CD38+HLA-DR+ CD4 T cells compared to the untreated group. In situations of chronic treated HIV, the presence of CD4 T cells expressing CPI molecules such as LAG-3 and PD1 may act to curb immune activation. In the ART naive viremic group on the other hand the CPI are not cardioprotective and immune activation is associated with increase in soluble markers of inflammation in plasma. Increased TNFRI, TNFRII, IL-6, IL-8, TNFα, LPS, and sCD14 were associated with lower cardiac function (data not shown).

Our study is limited by the fact that it is cross-sectional and we cannot provide direct evidence for increased LAG-3 or LAG-3 plus PD1 expressing CD4 T cells in the heart tissue leading to low cardiac function. In addition, we cannot formally show whether the increase in cells with these CPI molecules precedes the development of impaired cardiac function. Although we tried to adjust for all known risk factors, some other confounders may have existed among the study groups. Longitudinal studies are necessary to further explore and confirm the relationship of CPI LAG-3 and PD1 on cardiac function in HIV infected individuals on ART.

In conclusion, we found a novel association of LAG-3 expressing CD4 T cells either alone or in combination with PD1 with cardiac dysfunction in ART naïve viremic HIV+ patients, and with cardiac artery stiffness in both treatment naive and virally suppressed patients on ART. Key questions to investigate are 1) mechanism of action of LAG-3 for affecting the cardiac function and arterial structure, and 2) the basis that underlies synergies of LAG-3 with PD1 in regulating cardiac function. This understanding could provide insight into potential role of LAG-3 immunotherapy (which is in early clinical trials in cancer) in prevention or treatment of cardiac dysfunction in HIV.

## Supporting information

S1 Fig Soluble LAG-3 levels in plasma. sLAG3 in plasma were measured by ELISA in ART naive (Gp 1, n = 42), Virologically suppressed on ART Gp 2, (n = 21) and healthy controls (Gp 3, n = 21). Data compared between groups using Wilcoxon rank-sum test. A p value <0.05 was considered significant. ***p<0.001; ****p<0.0001.
(TIF)

S2 Fig Heatmap showing correlation of cardiac function and arterial stiffness related measures with LAG-3, PD1 and PD1+LAG-3+ CD4 T cells. Results shown for ART naïve (group 1,G1), and virologically suppressed on ART (group 2,G2) patients. Colored boxes represent significant (P < 0.05) correlation between analytes. Scale indicates the correlation coefficient with red color indicating the inverse correlations. For correlation analyses, Pearson correlation was performed based on data distribution. A *p* value of <0.05 was considered as significant as indicated by asterisk (*).
(TIF)
