# Supplementary figures and images for: Cardiac morbidity in HIV infection is associated with checkpoint inhibitor LAG-3 on CD4 T cells
Source: PLoS One. 2018 Oct 31;13(10):e0206256. doi: 10.1371/journal.pone.0206256 (PMC6209232; doi:10.1371/journal.pone.0206256)

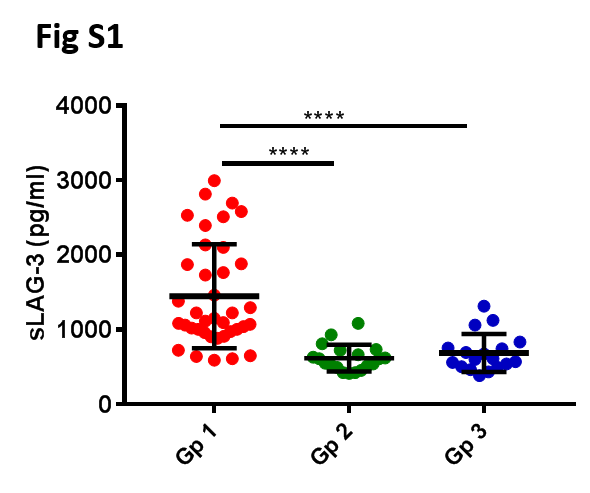

Supplement: S1 Fig — sLAG3 in plasma were measured by ELISA in ART naive (Gp 1, n = 42), Virologically suppressed on ART Gp 2, (n = 21) and healthy controls (Gp 3, n = 21). Data compared between groups using Wilcoxon rank-sum test. A p value <0.05 was considered significant. ***p<0.001; ****p<0.0001. (TIF) [file pone.0206256.s001.tif]

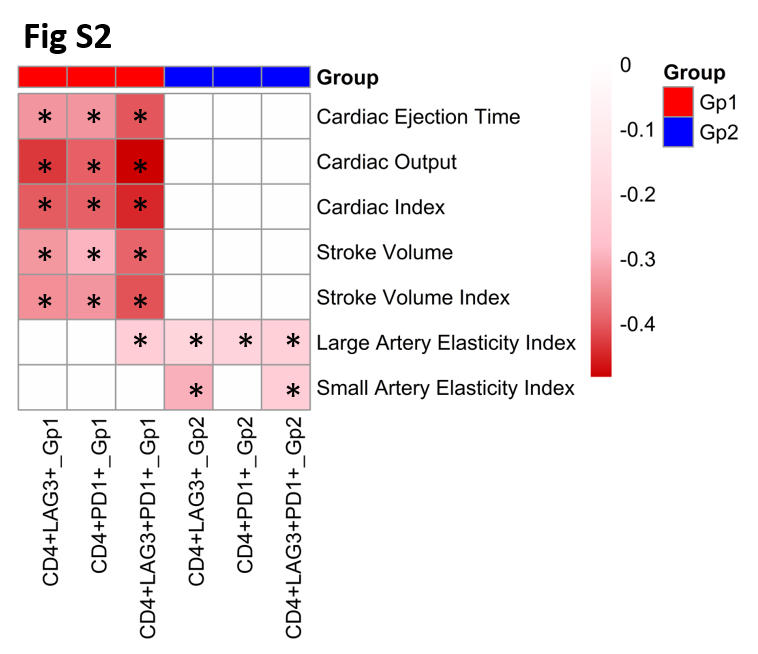

Supplement: S2 Fig — Results shown for ART naïve (group 1,G1), and virologically suppressed on ART (group 2,G2) patients. Colored boxes represent significant (P < 0.05) correlation between analytes. Scale indicates the correlation coefficient with red color indicating the inverse correlations. For correlation analyses, Pearson correlation was performed based on data distribution. A p value of <0.05 was considered as significant as indicated by asterisk (*). (TIF) [file pone.0206256.s002.tif]
